# An in-situ gas chromatography investigation into the suppression of oxygen gas evolution by coated amorphous cobalt-phosphate nanoparticles on oxide electrode

**DOI:** 10.1038/srep23394

**Published:** 2016-03-22

**Authors:** Jihyeon Gim, Jinju Song, Sungjin Kim, Jeonggeun Jo, Seokhun Kim, Jaegu Yoon, Donghan Kim, Suk-Gi Hong, Jin-Hwan Park, Vinod Mathew, Junhee Han, Sun-Ju Song, Jaekook Kim

**Affiliations:** 1 Department of Materials Science and Engineering, Chonnam National University, Gwangju 500-757, South Korea; 2 Energy Lab, Samsung Advanced Institute of Technology (SAIT), Samsung Electronics, Suwon 443-803, South Korea; 3 Department of Materials Science and Engineering, Korea Advanced Institute of Science and Technology (KAIST), Daejeon 305-701, South Korea

## Abstract

The real time detection of quantitative oxygen release from the cathode is performed by *in-situ* Gas Chromatography as a tool to not only determine the amount of oxygen release from a lithium-ion cell but also to address the safety concerns. This *in-situ* gas chromatography technique monitoring the gas evolution during electrochemical reaction presents opportunities to clearly understand the effect of surface modification and predict on the cathode stability. The oxide cathode, 0.5Li_2_MnO_3_∙0.5LiNi_0.4_Co_0.2_Mn_0.4_O_2_, surface modified by amorphous cobalt-phosphate nanoparticles (*a*-CoPO_4_) is prepared by a simple co-precipitation reaction followed by a mild heat treatment. The presence of a 40 nm thick *a*-CoPO_4_ coating layer wrapping the oxide powders is confirmed by electron microscopy. The electrochemical measurements reveal that the *a*-CoPO_4_ coated overlithiated layered oxide cathode shows better performances than the pristine counterpart. The enhanced performance of the surface modified oxide is attributed to the uniformly coated Co-P-O layer facilitating the suppression of O_2_ evolution and offering potential lithium host sites. Further, the formation of a stable SEI layer protecting electrolyte decomposition also contributes to enhanced stabilities with lesser voltage decay. The *in-situ* gas chromatography technique to study electrode safety offers opportunities to investigate the safety issues of a variety of nanostructured electrodes.

Since Li[Li_1/3−2*x*/3_Ni_*x*_Mn_2/3−*x*/3_]O_2_–type materials were first reported by Dahn and Ohzuku *et al.*, overlithiated layered oxides Li_1+*x*_M_1−*x*_O_2_ (M = Ni, Co, Mn, Cr, or combinations thereof) have attracted significant interest as potential alternatives to conventional cobalt and/or nickel-based cathode materials for high energy lithium-ion batteries because of their high capacity (≥200 mAh g^−1^), low-cost manganese (Mn) element, and high thermal stability in deeply charged states^1^,^2^,^3^,^4^,^5^,^6^,^7^,^8^,^9^. The layered-type structural characteristics of the overlithiated layered oxides Li_1+*x*_M_1−*x*_O_2_ (hereafter, denoted as OLO) facilitate the occupation of excess lithium ions amidst the transition metal layers^10^. The stoichiometric composition of these composite materials is also generally represented as “*x*Li[Li_1/3_Mn_2/3_]O_2_·(1−*x*)LiMO_2_” since the Li[Li_1/3_Mn_2/3_]O_2_−like region plays a decisive role in evaluating their structural stability and electrochemical characteristics during charge/discharge cycling. This specific notation also offers a convenient method of determining precursor molar concentrations to prepare OLO materials with a targeted stoichiometry.

OLO composites are generally represented as a combination of monoclinic (Li[Li_1/3_Mn_2/3_]O_2_ or Li_2_MnO_3_) and rhombohedral/trigonal (LiMO_2_) phases (or *x*Li[Li_1/3_Mn_2/3_]O_2_·(1−*x*)LiMO_2_). The monoclinic and rhombohedral phases are essentially identified by the corresponding major diffraction planes of (001) and (003), respectively, in the XRD patterns of OLO materials. The simultaneous presence of the corresponding major Bragg peaks clearly reveals that both the monoclinic and rhombohedral lattices with closely-packed layers are exquisitely integrated in the OLO composite cathodes^11^. The structure of OLO (or *x*Li[Li_1/3_Mn_2/3_]O_2_·(1−*x*)LiMO_2_) composites is similar to the well-known α-NaFeO_2_ layered structure belonging to the space group (SG) 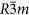 symmetry, except for a few additional Bragg peaks at scanning angles, 2θ = 20°–30°, which can be indexed to the Li[Li_1/3_Mn_2/3_]O_2_ phase (SG: C2/m). These XRD peaks present in the scanning angle (2θ) range between 20° and 35° are typically caused by the super-lattice ordering of Li^+^ and Mn^4+^ in the transition metal layer. In general, bulk Li[Li_1/3_Mn_2/3_]O_2_ possesses a layered structure with long range ordering of [Li_1/3_Mn_2/3_] in the transition metal layers so that well-resolved superstructure peaks are located between scanning angles (2θ) of 20° and 35°^12^. Furthermore, it has also been widely believed that bulk Li[Li_1/2_Mn_2/3_]O_2_, which has an ordered distribution of Li and Mn in the transition metal layers of the monoclinic lattice, is electrochemically inactive since the manganese oxidation state remains at Mn (IV) state so that no lithium can be deintercalated from this phase^12^.

However, investigations on nano-sized OLO composites *x*Li[Li_1/3_Mn_2/3_]O_2_·(1−*x*)LiMO_2_ revealed that the [Li_1/3_Mn_2/3_]O_2_ component tends to become electrochemically active beyond a cut-off potential of ~4.5 V, and thereby leads to Li_2_O formation, which in turn facilitates oxygen release as Li-extraction across the electrode occurs on deep charging. Thackeray *et al.* clearly explains the observed stoichiometry variation in a typical nano-sized OLO material (0.3Li[Li_1/3_Mn_2/3_]O_2_·0.7LiMn_0.5_Ni_0.5_O_2_) during electrochemical charge/discharge cycling^7^. The electrochemical activation of the monoclinic phase in OLO at high charging potentials is believed to enhance electrode performance and facilitate the realization of specific capacities as high as ~250 mAh g^−1^ within the voltage window of 4.6 (~4.8 V) and 3.0 V. Moreover, monoclinic Li[Li_1/3_Mn_2/3_]O_2_ appears to present a robust framework that not only prevents severe electrode degradation but also ensures the facile release of excess lithium during its decomposition as described in Equation 1 at high charging potentials and thereby contributes to enhanced electrode performances versus lithium^7^.


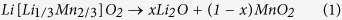


As illustrated by M. Thackeray, the initial voltage profile of OLO nanocomposites *x*Li[Li_1/3_Mn_2/3_]O_2_·(1−*x*)LiMO_2_ reveals a charge plateau at around 4.4 V^7^. Interestingly, this plateau is not observed in the subsequent charge cycles. The trend of the initial charge profile for OLO cathodes is mainly explained by two processes. Firstly, delithiation of the LiMO_2_ component occurs, concomitant with the oxidation of transition metal, M (M = Ni^2+/4+^, Mn^3+/4+^ or Co^3+/4+^). Secondly, as the OLO nanocomposite electrodes are cycled to high charging potentials (≥4.4 V), Li_2_O is extracted from the monoclinic Li[Li_1/3_Mn_2/3_]O_2_ component, followed by the release of chemical oxygen^7^,^12^. The unexpected removal of Li and O from the lattice of Li_2_MnO_3_ invariably results in the diffusion of transition metal ions from the surface to the core of the OLO nanoparticles^17^.

Therefore, many studies have centered on OLO cathode materials to understand the issues of gas evolution and its effect on electrode stability and safety during electrochemical reaction versus lithium. Furthermore, several efforts have been made to address the problems of capacity fade in OLO nanocomposite cathodes arising from the leaching out of transition metal ions at high charging potentials, especially under elevated temperatures^1^. Park *et al.* performed surface modifications on a typical OLO cathode *viz*. Li[Li_0.167_Ni_0.233_Co_0.100_ Mn_0.467_Mo_0.033_]O_2_ using Al_2_O_3_ and AlPO_4_ 18. The *in-situ* measurements of the internal cell pressure during electrochemical oxidation revealed that the considerable suppression of oxygen evolution led to significant improvement in the electrode stability during electrochemical reaction^18^. They also reported that wetting the fabricated cathode with vanadium precursor solution to facilitate the impregnation of VO_x_ into the layered structure also results in enhanced electrode stability^19^. Furthermore, several studies reported that surface modification of OLO nanocomposite cathodes with various materials such as self-catalyzed Polyaniline (PANI)^20^, lithium conductive Li_2_TiF_6_^21^, and Poly (3, 4-ehtylenedioxythiophene) Polystyrene sulfonate (PEDOT: PSS) led to the improvement of electrode performance and stability^22^.

Accordingly, many research groups have reported that introducing an amorphous oxide film on the surface of electrode particles, as a physical barrier, is a useful way to suppress the phenomenon of gas evolution. Supposing that the materials coated on the OLO are by nature potential lithium hosts, surface modification using amorphous materials may not only tend to trap O_2_ within the particle but also facilitate the hosting of excess lithium and thereby improve the cycle performances of OLO. Transition metal phosphates (MPO_4_), in general, are good candidates useful for coating on unstable electrode materials due to their structural stability and ability to serve as lithium intercalation hosts during electrochemical discharge cycling. Among these, cobalt-phosphate is attractive for coating because its working potential is about 4.8 V, which is very close to the potential (~4.5 V) of Li_2_MnO_3_ decomposition and the associated oxygen gas evolution. Further, CoPO_4_ will remain active towards lithium reactivity at this same potential and can tend to contribute to specific capacities. In the light of the above discussions, the present work reports the effect of an amorphous cobalt-phosphate (*a*-CoPO_4_) coating film on an OLO nanocomposite cathode, namely 0.5Li_2_MnO_3_·0.5Li[Ni_0.4_Co_0.2_Mn_0.4_]O_2_ in diminishing voltage decay issue as cycled. More importantly, the real time detection of quantitative oxygen release in this nanocomposite cathode is performed by employing *in-situ* Gas Chromatography (GC) as a tool to not only determine the amount of oxygen release from a lithium-ion cell but also to address the safety concerns of utilizing OLO-based cathodes in lithium-ion battery applications.

## Results

**Figure 1:**
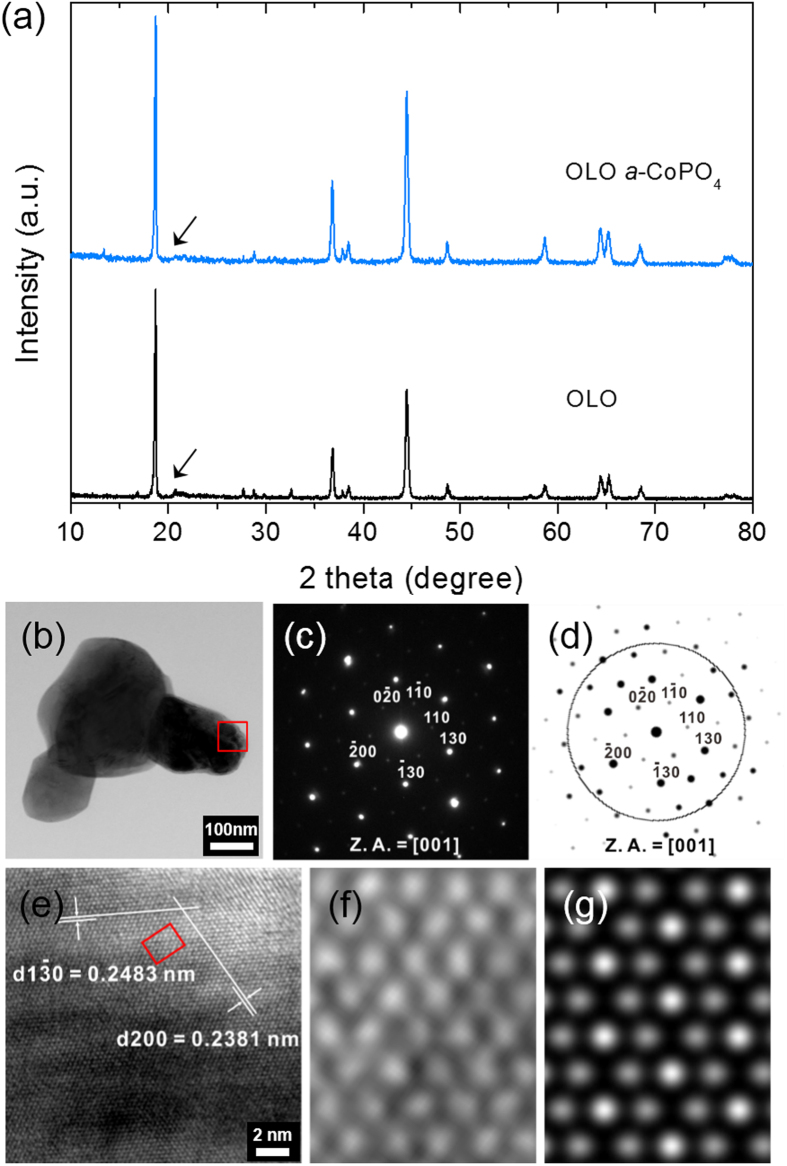
(**a**) X-ray diffraction (XRD) patterns of reference OLO and *a*-CoPO_4_ coated OLO, (**b**) Bright-field TEM image of OLO particles, (**c**) its corresponding SAED pattern projected on [001] zone axis, (**d**) calculated diffraction pattern with the same zone axis as that of (**c**), (**e**) High-resolution (HR) TEM image of OLO particle taken from red square region in (**b**), (**f**) magnified HR TEM image taken from red rectangular region and (**g**) its simulated lattice image.

The X-ray diffraction (XRD) patterns of pristine and *a*-CoPO_4_ coated 0.5Li_2_MnO_3_∙0.5LiNi_0.4_Co_0.2_Mn_0.4_O_2_ are shown in Fig. 1a. The XRD patterns of both the pristine and surface modified samples are well indexed to the rhombohedral phase with R
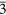
m space group. The minor peak traces in the scanning angle (2θ) ranging between 20° and 23.5° (earmarked by an arrow in Fig. 1a) are attributed to the cation ordering of the lithium-transition metal layer (LiM_6_) plane and are referred to as ‘super lattice peaks’ indexed to the C2/m symmetry of the monoclinic Li_2_MnO_3_ phase^4^,^5^,^7^,^24^. The fact that little or no variation is observed in the XRD patterns of both the pristine OLO and *a*-CoPO_4_ coated OLO not only reveals that the overall crystal structure of OLO is retained in both samples, but also suggests that *a*-CoPO_4_ is present as an amorphous phase without influencing any structural change. In order to understand the contents of the amorphous phase, CoPO_4_ was prepared separately using the same precursor concentrations by solid-state reaction at 800 °C in air. The XRD results of the final product reveal the presence of the relatively oxygen deficient phase of Co_2_P_2_O_7_ and the diffraction lines are well matched with the simulated pattern of ICSD#203161 (see Supplementary Fig. S1). The observed results thus indirectly suggest that the amorphous CoPO_4_ formed/coated on the surface of the OLO particles is most likely to be composed of CoPO_4_ and Co_2_P_2_O_7_ phases. Literature reported that the crystal structure of CoPO_4_ is unstable in air and on heating at 350 °C the formation of Co_2_P_2_O_7_ is inevitable due to the oxygen release from Co^3+^PO_4_ and structural rearrangement^25^. Therefore, it is anticipated that the amorphous CoPO_4_ coating layer can be composed of both CoPO_4_ and Co_2_P_2_O_7_-like phases, simultaneously.

The bright-field transmission electron microscope results (BF-TEM) of the pristine sample are shown in Fig. 1b. The selected area electron diffraction (SAED) pattern (of the earmarked rectangle with red border in Fig. 1b) projected on the [001] zone axis (Fig. 1c) reflects the triplet dark spots indicating the presence of Li_2_MnO_3_-like domains and the ordering of lithium ions with TM ions in TM layers^26^. As expected, the calculated/simulated diffraction pattern (Fig. 1d) obtained using the zone axis of [001] is in agreement with the observed pattern shown in Fig. 1c. The high resolution TEM (HR-TEM) image in Fig. 1e reveals distinguishable lattice fringe distances of 0.2483 nm and 0.2381 nm that correspond to the (1
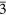
0) and (200) diffraction planes, respectively, of monoclinic Li_2_MnO_3_. It is very worthy to note that the overall atomic arrangement in the OLO structure is observed as a suspended mixture of both rhombohedral and monoclinic phases without specific boundaries while the transition metal layer is generally represented by a nanocomposite structure with intimate mixture of both phases showing specific regional arrangement. It is reasonable to expect that the decomposition reaction of Li_2_MnO_3_ phase might be initiated at the early charging state due to the pre-activated lithium originated from structural properties similar to nano-sized Li_2_MnO_3_^23^. The magnified atomic arrangement in the OLO structure (corresponding to the red rectangular region Fig. 1e) shown in Fig. 1f is identical with the simulated lattice image (Fig. 1g) under a thickness of 206 nm and a defocus condition of −12 nm. These observations thus confirm the solid solution characteristics of LiMO_2_ and Li_2_MnO_3_ phases in OLO-type materials. Further, the TEM results confirm the finding from the XRD studies (Fig. 1a) that the minor peaks around the scanning angle (2θ) of 20° indicate the cation ordering of the LiM_6_ plane in the Li_2_MnO_3_ phase of the prepared OLO material.

**Figure 2:**
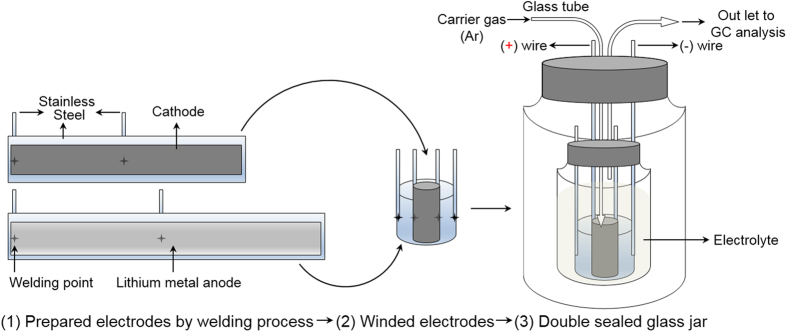
*In-situ* GC cell fabrication for GC analysis detecting O_2_ gas release.

In order to evaluate the electrode stability, *in-situ* gas chromatography (GC, Aglient Technology; 6890N, Network GC System) investigations were performed on specially designed test half-cells (Fig. 2) fabricated using the prepared OLO electrodes by measuring the quantitative amount of oxygen gas evolved from the cathode during electrochemical oxidation. During measurements, the test cells were maintained under a constant flow of inert argon gas (carrier gas) to ensure that the gases evolving from the electrode immersed in the electrolyte drifted to the headspace from where they are pumped off via a capillary to the gas chromatography system.

On completion of the gas evolution measurement for the fabricated cells, the output chart of the GC system indicated that a mixture of H_2_, O_2_, N_2_, and Ar gases represented by separate consecutive columns versus time dependency were detected^27^. These results indicate that the GC measurements can also be used for the detection of H_2_ gases in any electrode that requires to be analyzed for structural stability^11^,^28^. However, the focus of the present study is to just measure the evolved oxygen gas from the GC measurement and thereby evaluate the stability of the prepared layered- OLO cathodes.

**Figure 3:**
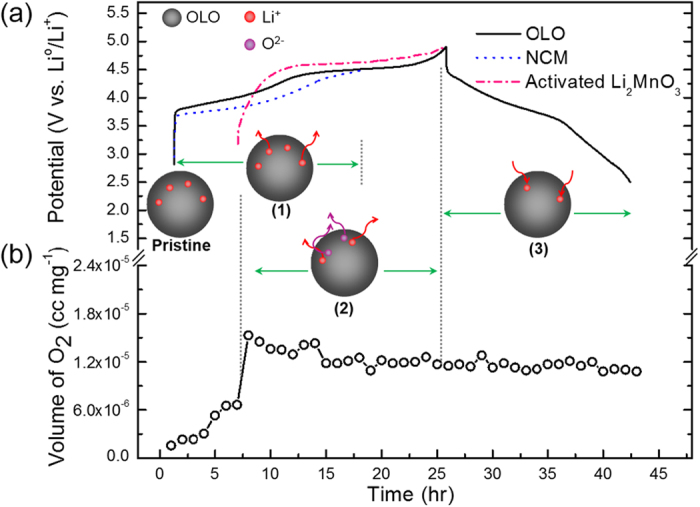
*In-situ* GC results of O_2_ gas evolution plotted as a function of charging time and the proposed mechanism of gas evolution in the pristine OLO electrodes based on (**a**) their initial voltage profiles comparing to commercial layered NCM electrode (blue dot) and activated nano-sized Li_2_MnO_3_ electrode^25^ (pink dash dot), and (**b**) amount of oxygen gas as a function of charging step potentials for the pristine OLO electrodes.

The real time plots drawn between the oxygen gas volume versus charging step potentials and time period in Fig. 3 indicate that the chemical oxygen is evolved during charge cycling in the pristine electrode. Based on this observation, the underlying mechanism for gas evolution related to the decomposition of Li_2_MnO_3_ in the pristine electrode can be explained using their respective initial voltage profile shown in Fig. 3a. In order to ascertain the exact time period of electrode activation occurring in the prepared electrodes, a very low current density was maintained during the GC measurements. The pristine OLO electrode tends to release oxygen even at lower charging potentials, as observed from the oxygen volume *vs*. charging time plot, due to the pre-activated lithium ion related to the solid solution characteristics of the prepared OLO. For further understanding, the typical voltage profiles of layered oxides, LiNi_0.333_Co_0.333_Mn_0.333_O_2_ (NCM), and nano-sized Li_2_MnO_3_ are also provided for comparison purposes in Fig. 3a. On comparison with the typical electrochemical charge curve of NCM, the decomposition of Li_2_MnO_3_ in the present OLO should likely occur at high charging potentials (>4.45 V) as is characteristic of OLO cathodes. However, on comparison with the characteristic profile of nano-sized Li_2_MnO_3_ in Fig. 3a, the oxygen gas release observed for the present OLO electrode at lower charging potentials (<4.45 V, in Fig. 3b) may be attributed to the activated Li_2_MnO_3_ characteristics^23^. Also, this specific phenomenon during early lithium extraction can be well explained using the comparative charge profiles provided in Fig. 3a. Precisely, the charging curve of the pristine OLO electrode at the early stage is slightly higher than that for the NCM electrode (blue dot line), and possibly corresponds to the reaction occurring in activated Li_2_MnO_3_ (pink dash dot line). This implies that lithium extraction from the Li_2_MnO_3_-type phase suspended in the present OLO (as observed from the TEM results in Fig. 1) may contribute to the rising curve at lower charge potentials. The observed results thus suggest that this oxygen gas release can be suppressed in the present OLO by modifying its surface using appropriate materials.

**Figure 4:**
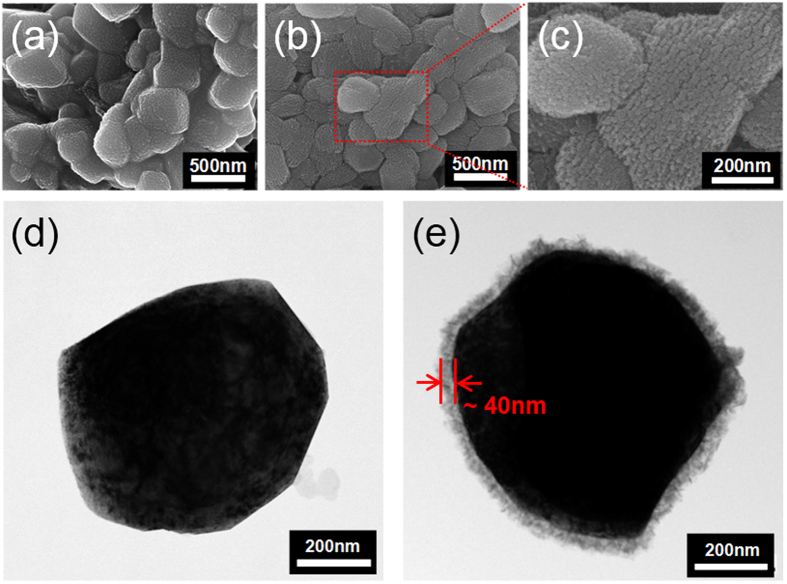
SEM images of (**a**) pristine OLO, (**b**) *a*-CoPO_4_ coated OLO, and (**c**) magnified SEM image of (**b**). HR-TEM images of (**d**) pristine OLO and (**e**) *a*-CoPO_4_ coated OLO.

Field-Emission SEM was used for the observation of the particle morphology of the prepared pristine OLO (Fig. 4a) and *a*-CoPO_4_ coated OLO (Fig. 4b). A magnified SEM image (Fig. 4c) is shown to clarify the coating layer of amorphous cobalt-phosphate. The morphology of pristine OLO shown in Fig. 4a reveals aggregated quasi-spherical particles with sizes in the range of 250–500 nm; the surface of the OLO particles appear to be quite rough, probably due to the irregular thermal decomposition of oxalate and acetate-based organic compounds. However, although the particle-size variations are not observable, the surface on the *a*-CoPO_4_ coated OLO particles appear to differ from that of the pristine sample and reveals an almost uniform coating layer on the particles, as shown in Fig. 4b.

**Figure 5:**
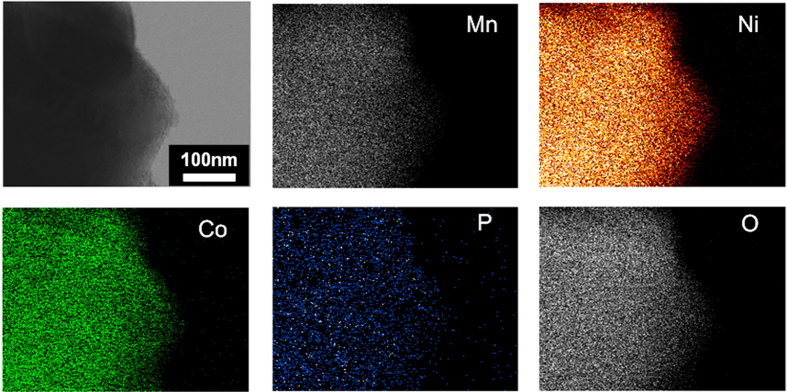
EDAX elemental mapping of Mn, Ni, Co, P and O elements for *a*-CoPO_4_ coated OLO sample by scanning TEM (STEM).

Furthermore, the magnified image in Fig. 4c reveals that the coated layer of amorphous CoPO_4_ appears to form a worm-like morphology on the surface of the OLO particles. For further confirmation of the surface coating, HR-TEM images of the pristine and *a*-CoPO_4_ coated OLO were recorded and the results are shown in Fig. 4d and Fig. 4e, respectively. The image of the pristine sample (Fig. 4d) shows an OLO particle devoid of any surface modification, whereas the image of the surface coated sample (Fig. 4e) clearly reveals the presence of an amorphous layer of 40 nm thickness on the particle surface. To elucidate further, elemental mapping using STEM was performed to analyze the distribution of chemical elements in the coated sample and the results are presented in Fig. 5. The STEM mapping images in Fig. 5 reveal a uniform distribution of Co, P, and O elements in the area under study and thereby not only suggest the presence of amorphous CoPO_4_ in the OLO particles but also demonstrate that the *a*-CoPO_4_ coating layer is uniformly spread on the surface of the OLO particles, as observed from the HR-TEM studies. It is reasonable to conclude that the surface coating observed as a well wrapped layer around the particle, as shown in Fig. 4e, might act as a physical barrier to ultimately suppress the oxygen gas evolution from the OLO cathode.

**Figure 6:**
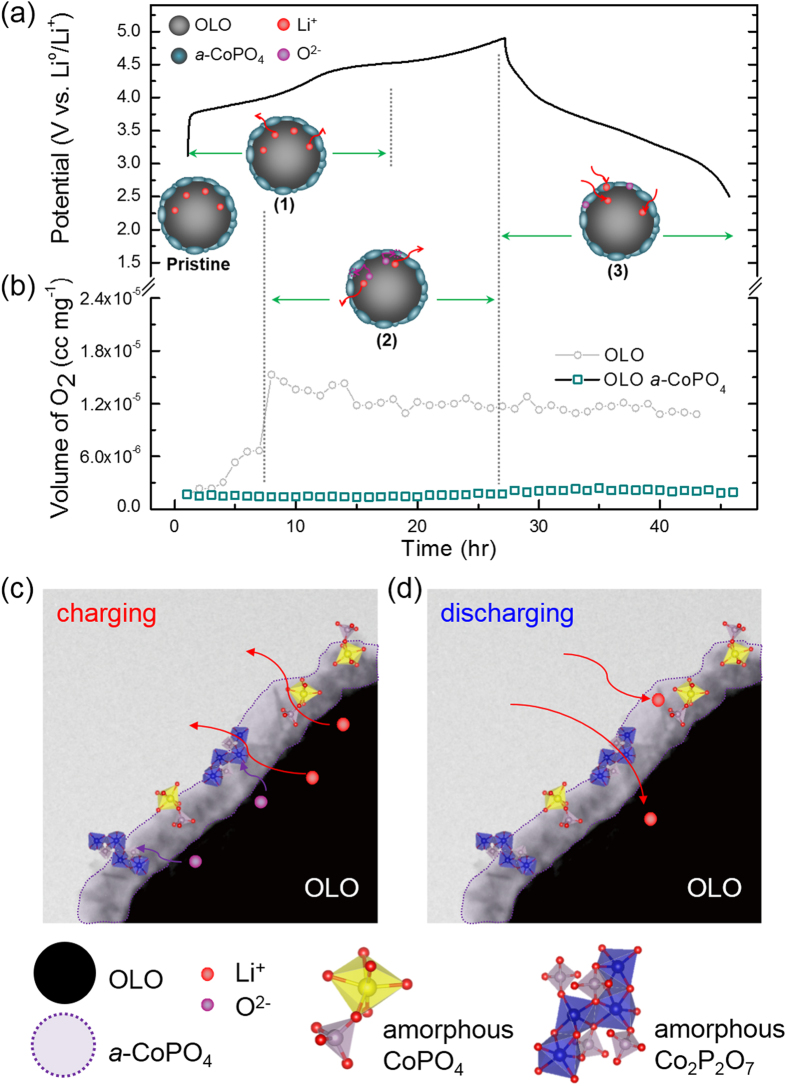
*In-situ* GC results of O_2_ gas evolution plotted as a function of charging time based on (**a**) their initial voltage profiles and (**b**) amount of oxygen gas as a function of charging step potentials for the pristine OLO and *a*-CoPO_4_ coated OLO electrodes. A schematic that explains the mechanism for oxygen gas suppression and the electrochemical reaction with lithium during (**c**) charging and (**d**) discharging of the *a*-CoPO_4_ coated OLO cathode.

The *in-situ* GC measurement was performed on the galvanic test cell with amorphous CoPO_4_ coated OLO cathode to confirm the effect of surface modification and the results are shown in Fig. 6. The comparative time versus oxygen volume plots in Fig. 6b clearly reveals that oxygen release is significantly suppressed and thereby leads to higher specific capacities in the surface modified OLO cathode (Fig. 6a). In addition, the characteristic of Co^2+/^Co^3+^ redox couple transition in crystalline CoPO_4_ is known to occur at 4.8 V vs. Li^0^/Li^+^. Hence, it is highly possible that Li-intercalation into *a*-CoPO_4_ nanoparticle during electrochemical reduction may also contribute to higher discharge capacities^29^. Consequently, the electrochemical reaction mechanism (Fig. 6c,d) in the *a*-CoPO_4_ coated OLO during charge and discharge cycling can be explained in three major stages: (1) lithium de-intercalation from the rhombohedral phase of layered oxide, LiNi_0.4_Co_0.2_Mn_0.4_O_2_ under low charging potentials (<4.45 V), (2) lithium removal due to the decomposition of monoclinic Li_2_MnO_3_ phase and the trapping of oxygen (suppression of oxygen evolution) by the *a*-CoPO_4_ wrapped layer acting as a physical and chemical barrier at deep charging potentials due to the characteristic properties of numerically oxygen deficient Co_2_P_2_O_7_ phase, and (3) simultaneous lithium intercalation into OLO and reaction with the *a*-CoPO_4_ coating layer in the consecutive discharge cycling due to the action of CoPO_4_−like materials as potential lithium hosts. The present GC analysis therefore provides not only direct visual evidence for oxygen evolution at high charging potentials in OLO-based cathodes, but also provides evidence that an amorphous Co-P-O coated layer on the surface of OLO might play the role of a buffer layer and thereby effectively suppress O_2_ gas evolution. In addition, the GC analysis demonstrated in the present study may help to identify potential OLO hosts that can be surface modified by a variety of amorphous oxide/phosphate films. Furthermore, the *in-situ* GC technique presented here may facilitate the understanding of the phenomenon related with oxygen gas evolution in a variety of surface modified OLO-based cathodes and thereby predict their electrode stability for potential lithium battery applications. In order to further study the effect of amorphous CoPO_4_ coating layer on the electrochemical activity of the electrode, the electrochemical impedance spectroscopy (EIS) was used to investigate the role of amorphous-CoPO_4_ coating layer by comparing the pristine OLO to *a*-CoPO_4_ coated OLO cathodes. The Nyquist plots fitted using the Non-Linear Square fit method in the ZView program and the corresponding equivalent circuits for the pristine OLO and *a*-CoPO_4_ coated OLO cells at OCV (fresh cell) and 1^st^ fully charged stage are provided in the Supplementary Information (see supplementary Fig. S3). The semicircle in high-medium frequency is ascribed to the charge-transfer resistance (R_ct_), which represents the lithium ion migration between the electrode and electrolyte^30^. By comparing the R_ct_ values, it is found that the charge-transfer resistance of *a*-CoPO_4_ coated OLO at OCV is relatively higher than pristine OLO electrodes, indicating that the coating layer might be acting as a electrochemically insulating film similar to that of a stable SEI layer. However, the R_ct_ value of *a*-CoPO_4_ coated OLO was dramatically decreased during charging and revealed a smaller value than that of pristine OLO at fully charged state as shown in Supplementary Fig. S3b. The decreased impedance is most probably attributed to the possible electrochemical reaction of lithium ion with the amorphous CoPO_4_ coating during the time interval between consecutive alternating current potentials applied in steps of 10 mV. Therefore, the trend of the observed EIS results appear to reflect the effect of *a*-CoPO_4_ coating in OLO and indirectly supports the conclusion that the higher capacity achieved is related to the surface modification of OLO by *a*-CoPO_4_.

**Figure 7:**
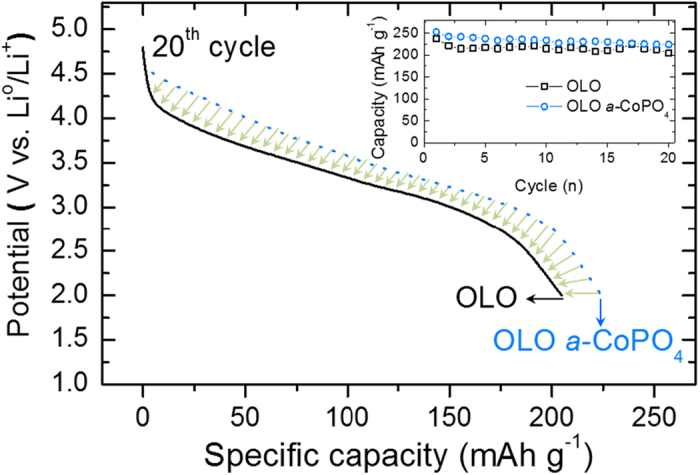
Discharge profiles at the 20^th^ cycle in the voltage range of 2.0–4.8 V for pristine OLO (black solid) and *a*-CoPO_4_ coated OLO (blue dot) and their cycleabilities as inset.

Figure 7 provides the comparison of the 20^th^ discharge profiles for untreated OLO and surface coated OLO cathodes within the potential window of 2.0–4.8 V *vs*. Li^0^/Li^+^ at a current density of 14.3 mA g^−1^. Surprisingly, the degradation is significantly improved as indicated by the green arrows in Fig. 7. In addition, the detailed initial and 20^th^ voltage profiles of the pristine and amorphous CoPO_4_ coated OLO electrodes are provided in Supplementary Fig. S2. As anticipated, the charge capacity contribution below 4.4 V is attributed to the deintercalation of lithium from layered LiMO_2_ (here, LiNi_0.4_Co_0.2_Mn_0.4_O_2_) for both electrodes. On the other hand, the long charge plateau observed beyond 4.4 V (see Supplementary Fig. S2) arises from the electrochemical decomposition of Li_2_MnO_3_ to Li_2_O and MnO_2_ accompanied with the inevasible oxygen gas release resulting from the electrode decomposition as shown in Figs 3a and 6a4,^31^. The initial voltage profiles of the pristine and *a*-CoPO_4_ coated OLO electrodes appear to be similar; however, the charge and discharge capacities obtained for the surface modified OLO (365 and 252 mAh g^−1^) are 13% and 9% higher than that observed for the pristine OLO electrode (320 and 237 mAh g^−1^), respectively (see Supplementary Fig. S2).

Although the Coulombic efficiency of 69% for the *a*-CoPO_4_ coated OLO is lower than that of 74% for the pristine OLO, the higher discharge value in surface modified OLO is realized most probably due to its higher lithium reactivity arising from the presence of potential lithium-host materials (Fig. S2). It is well known that dissolution of transition metals in high voltage is a drawback and an inevitable phenomenon due to the chemical attack of HF arising from electrolyte decomposition. However, in the present case, the amorphous coating layer can serve as a stable SEI layer, which can preserve the surface of active materials thereby stimulating electrochemical reaction with the decomposition of Li_2_MnO_3_ to Li_2_O and MnO_2_ and contribute to higher charge capacity than bare OLO. Hence, it is reasonable to expect that charge capacity of *a*-CoPO_4_ coated OLO is higher than that of pristine OLO due to the stable surface coating layer that tends to hinder a chemical attack due to electrolyte decomposition in the former. In addition, the higher discharge capacity of *a*-CoPO_4_ coated OLO can be reasonably attributed to the capacity contribution from possible lithium-sites in the coated CoPO_4_ layer. The inset (top right) in Fig. 7 compares the cycleability of both electrodes under the same current density of 14.3 mA g^−1^. The discharge capacity of both electrodes decreased gradually during extended cycling. Pristine OLO electrode shows significant capacity fading as only 86.1% capacity is retained compared to 88.8% for *a*-CoPO_4_ coated OLO in the 20^th^ cycle. It is highly likely that the slightly high capacity retention capability of the surface modified OLO originates from the reduction of electrolyte decomposition due to the protective coating layer of *a*-CoPO_4_ on the surface of OLO particles. The phenomenon of voltage decay and degradation of electrodes in fact are very typical electrochemical trends for high manganese based lithium rich materials due to their structural transformation to spinel like structure, thereby lowering energy density during extended cycling^22^,^32^. The ex-situ STEM EDAX (see Supplementary Fig. S4) was performed to confirm the stability of coating layer after the 50^th^ discharge cycle. The elemental mapping images reveal a uniform distribution of Co, P, and O and are similar to that observed for the case for the parent/as-prepared *a*-CoPO_4_ coated OLO (before electrochemical measurement and shown in Fig. 5). From these results, it can be assumed that the coated amorphous cobalt-phosphate layer is rigidly maintained with no significant surface damages thereby contributing to the stability of OLO materials by oxygen gas suppression and facilitating enhanced electrochemical properties. Therefore, it is reasonable to conclude that the amorphous CoPO_4_ coating layer plays the role of an oxygen buffer layer as well as a plausible lithium-host upon prolonged cycling.

**Figure 8:**
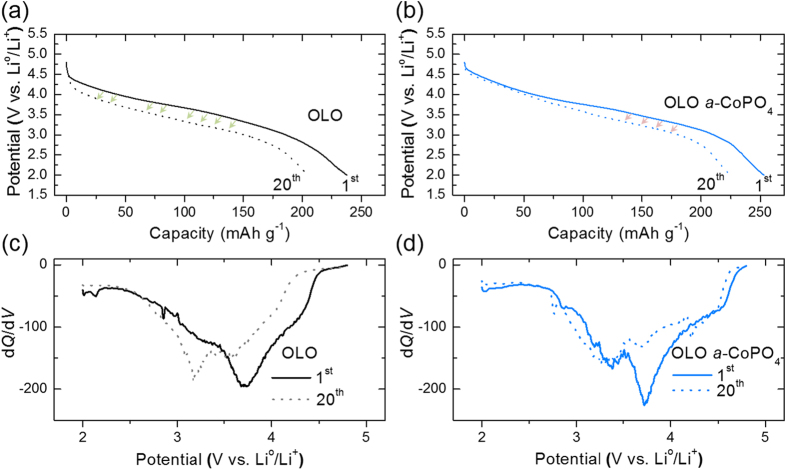
Discharge profiles at the 1^st^ and 20^th^ cycle in the voltage range of 2.0–4.8 V for (**a**) pristine OLO, (**b**) *a*-CoPO_4_ coated OLO, and the d*Q*/d*V* plots derived from the initial and 20^th^ cycle curves for (**c**) OLO and (**d**) *a*-CoPO_4_ coated OLO, respectively.

Further, the visual proof of the electrochemical improvement of surface modification with amorphous CoPO_4_ can be observed by comparing the differential capacity (d*Q*/d*V*) plots deduced from the 1^st^ and 20^th^ discharge profiles (Fig. 8b) of the pristine and surface-treated OLO electrodes, respectively. It is worthy to note that the reduction (d*Q*/d*V*) peak observed for pristine OLO shifted from 3.8 V in the 1^st^ cycle to 3 V in the 20^th^ cycle, as observed from Fig. 8c whereas the *a*-CoPO_4_ coated OLO cathode shows negligible shift from the reduction peak observed at 3.8 V in the 1^st^ and the 20^th^ cycle (Fig. 8d). The noticeable shift in the peak position observed for pristine OLO towards lower discharge potentials suggests a significant voltage decay phenomenon that mainly arises from the phase transformation to spinel-like structure. Overall, the superior electrochemical stability of OLO surface coated with *a*-CoPO_4_ is attributed to the amorphous coating layer that can serve as a trap for the oxygen released during Li_2_MnO_3_ decomposition, a protection from severe electrolyte decomposition and a plausible capacity contribution from *a*-CoPO_4_ as a potential lithium-host.

## Discussion & Conclusion

An amorphous cobalt-phosphate (*a*-CoPO_4_) coated OLO (0.5Li_2_MnO_3_∙0.5LiNi_0.4_Co_0.2_Mn_0.4_O_2_) cathode was prepared by a simple precipitation reaction followed by mild heat treatment at 350 °C. The electron microscopy studies confirmed that the OLO nanoparticle was wrapped in an amorphous CoPO_4_ layer of 40 nm thickness. The electrochemical performance of the pristine OLO and surface modified or *a*-CoPO_4_ coated OLO cathodes versus lithium were investigated. The increased discharge capacities of *a*-CoPO_4_ coated OLO were attributed to the stable SEI layer protecting the electrolyte decomposition during the electrochemical reaction. The surface modification of OLO by an amorphous Co-P-O layer on the surface facilitates the suppression of O_2_ gas evolution during charge cycling and this study revealed the effect of coating on the improvement of electrode stabiliy from *in-situ* GC analysis. Therefore, the enhanced electrochemical activity of *a*-CoPO_4_ OLO can be attributed to the protective amorphous Co-P-O coating layer (similar to the protective SEI-like layer) from electrolyte decomposition and a plausible capacity contribution from *a*-CoPO_4_ as a potential lithium-host.

In conclusion, the study presents an *in-situ* GC technique that not only aids in monitoring the gas evolution occurring in OLO-based cathodes during electrochemical oxidation but also presents opportunities to clearly understand the effect of surface modification on OLO-cathodes with respect to gas evolution at higher potentials and their role in analyzing the stability and performance of such cathodes versus lithium. It is worth noting that the *in-situ* GC analysis of surface modified electrodes represent a reasonable technique for the evaluation of electrode stability. Furthermore, the mechanism may be more clearly understood if the local structural information is obtained by utilizing various analytic methods.

## Methods

### Material synthesis

Appropriate amounts of lithium acetate, nickel acetate, cobalt acetate, and manganese acetate approximate to the target stoichiometry of 0.5Li_2_MnO_3_∙0.5LiNi_0.4_Co_0.2_Mn_0.4_O_2_ were dissolved in deionized water. The resulting solution was left under stirring at 40 °C until the precursors were completely dissolved. Oxalic acid solution was then poured into the homogenous solution and precipitates formed after a few seconds. After evaporation of the water on the hotplate overnight, the precipitates were decomposed at 500 °C for 3 h and subsequently calcined at 900 °C for 12 h in air to obtain the reference OLO material. The sample prepared thus was provided by the Samsung Advanced Institute of Technology (SAIT). Stoichiometric amounts of cobalt acetate and ammonium dihydrogen phosphate were dissolved in distilled water with dispersed reference OLO powder for the surface modification of 3wt% of amorphous cobalt-phosphte. On completion of the reaction, the resulting precipitate was dried at 80 °C in a muffled oven before heating at 350 °C for 5 h to obtain the final CoPO_4_ surface-coated OLO powder. For comaprison purposes, the activated Li_2_MnO_3_ with nano-sized particles was prepared by oxidation reaction, as reported in our earliar literature^23^ and LiNi_1/3_Co_1/3_Mn_1/3_O_2_ (NCM) material was provided by Daejung Energy Materials Co., LTD.

### Material characterization

The powder X-ray diffraction data of the samples were recorded using a Shimadzu X-ray diffractometer with Ni-filtered Cu Kα radiation (λ = 1.5406 Å) operated at 40 kV and 30 mA within the scanning angle, 2θ, range of 10°–80° in steps of 0.01°. The surface morphologies and particle size of the obtained powders were examined by field-emission scanning electron microscopy (FE-SEM, S-4700 Hitachi) and field-emission transmission electron microscopy (FE-TEM, Philips Tecnai F20 at 200 kV in KAIST), along with corresponding selected-area electron diffraction (SAED) patterns. For FE-TEM, powder samples were ultrasonically dispersed in ethanol, and a few drops were coated onto copper grids. FE-TEM energy dispersive x-ray spectrometry (EDAX) was conducted to confirm the coating layer. Mapping of the distribution of the different chemical elements constituting the specimen was obtained by EDAX analysis. Ex-situ TEM EDAX was performed on the electrodes recovered from the test cell after 50 cycle measurements. The cathode was removed from the cycled cell using a coin-cell disassembler. The obtained cathode was washed by DMC solvent for eliminating residual lithium salts or other contaminants for precise observation of TEM images. The electrode was detached from current collector and dispersed in ethanol with high-energy ultrasonication (Ultrasonic processor with a tip diameter of 3 mm, SONICS, USA) before TEM observation.

### Electrochemical measurements

Electrochemical measurements were performed on coin-cells (type 2032) using Li foil as the counter electrode. The working electrodes were fabricated using a specific weight ratio of 75:10:15 (wt. %) of active materials, conductive carbon (ketjen black), and polytetrafluoroethylene (PTFE) binder, respectively. The mixture electrode was pressed onto a stainless steel mesh of 16 mm diameter with loading density of 3.4 mg cm^−1^ and vacuum dried at 120 °C for 12 h, thus forming the cathode. A 2032 coin type cell consisting of the cathode and lithium metal anode separated by a polymer membrane together with glass fiber was fabricated in an Ar-filled glove box and aged for 12 h before conducting the electrochemical measurements. The electrolyte of 1M LiPF_6_ was dissolved in a mixture of ethylene carbonate and dimethyl carbonate (1:1 volume ratio). Galvanostatic testing (BTS–2004H, Nagano, Japan) of the coin cells was conducted using a programmable battery tester over the potential range of 2.0–4.8 V vs. Li^0^/Li^+^. The Electrochemical Impedance Spectroscopy (EIS) measurement of the electrodes were carried out on VSP-5 Potentiostat/Galvanostat with EIS system (Bio-Logic, SAS, FRANCE) in the frequency range from 0.04 Hz to 1.0 M Hz with AC signal of 10 mV perturbation.

### *In-situ* gas chromatography studies

The electrode system newly designed for the measurement of oxygen detection is shown in Fig. 2. The working electrodes were prepared with the weight ratio of 85:5:10 (wt. %) of active materials, cunductive carbon and polyvintlidene fluoride (PVDF) binder. A hollow-type cylinder cathode was fabricated using a stainless steel strip as the collector, and a lithium metal foil was rolled into another hollow-type cylinder of larger diameter and was used as the anode. The prepared electrode system was sealed with silicone gel in a small bottle containing a sufficient amount of electrolyte. This bottle was then enclosed in a larger air-tight bottle and sealed to maintain minimum air exposure. The prepared galvanic cell system was then cycled and tested using *in situ* gas chromatography (GC, Agilent Technologies 6890 N) with a thermal conductivity detector (TCD) in Ar stream as a carrier gas with a flow rate of 30 cc min^−1^.

## Additional Information

**How to cite this article**: Gim, J. *et al.* An in-situ gas chromatography investigation into the suppression of oxygen gas evolution by coated amorphous cobalt-phosphate nanoparticles on oxide electrode. *Sci. Rep.* **6**, 23394; doi: 10.1038/srep23394 (2016).

## Supplementary Material

Supplementary Information
