# Supplementary material for: An in-situ gas chromatography investigation into the suppression of oxygen gas evolution by coated amorphous cobalt-phosphate nanoparticles on oxide electrode
Source: Sci Rep. 2016 Mar 22;6:23394. doi: 10.1038/srep23394 (PMC4802213; doi:10.1038/srep23394)
Supplement: Supplementary Information [file srep23394-s1.doc]

**Supplementary Information**

**An in-situ gas chromatography investigation into the suppression of oxygen gas evolution by coated amorphous cobalt-phosphate nanoparticles on oxide electrode**

Jihyeon Gim^1†^, Jinju Song^1†^, Sungjin Kim^1^, Jeonggeun Jo^1^, Seokhun Kim^1^, Jaegu Yoon^2^, Donghan Kim^2^, Suk-Gi Hong^2^, Jin-Hwan Park^2^, Vinod Mathew^1^, Junhee Han^3^, Sun-Ju Song^1^, and Jaekook Kim^1^*

^1^Department of Materials Science and Engineering, Chonnam National University, Gwangju 500-757, Republic of Korea.

^2^Energy Lab, Samsung Advanced Institute of Technology (SAIT), Samsung Electronics, Suwon 443-803, Republic of Korea

^3^Department of Materials Science and Engineering, Korea Advanced Institute of Science and Technology (KAIST), Daejeon 305-701, Republic of Korea

^†^These authors contributed equally to this work.

^*^Corresponding author. Tel: +82-62-530-1703. Fax: +82-62-530-1699.

E-mail: [jaekook@chonnam.ac.kr](mailto:jaekook@chonnam.ac.kr) (Jaekook Kim)

**Figure S1.** X Ray Diffraction (XRD) pattern of cobalt phosphate prepared by solid-state reaction at 800 ºC in air atmosphere and the simulated pattern.

Cobalt acetate and ammonium dihydrogen phosphate precursors corresponding to the same molar ratio (used in the wet preparation of surface modified OLO) were mixed in the solid-state and heated at 800ºC in air in order to indirectly detect the precise phase formed during the wet chemical route followed for coating of the OLO. The XRD pattern of the final product after solid-state reaction appears to match well with the simulated pattern of Co_2_P_2_O_7_ (ICSD # 203161).

**Figure S2.** Initial charge/discharge curve profiles of pristine OLO and *a*-CoPO_4_ coated OLO cathodes.

**Figure S3.** The Nyquist plots of both pristine OLO and *a*-CoPO_4_ coated OLO at (a) OCV and at (b) 1^st^ charged state along with the equivalent circuit in the inset.

**Figure S4.** Ex-situ study of EDAX elemental mapping of Mn, Ni, Co, P and O for *a*-CoPO_4_ coated OLO after 50 cycles.
